# Automated patient positioning in CT using a 3D camera for body contour detection: accuracy in pediatric patients

**DOI:** 10.1007/s00330-020-07097-w

**Published:** 2020-08-04

**Authors:** Ronald Booij, Marcel van Straten, Andreas Wimmer, Ricardo P.J. Budde

**Affiliations:** 1 Department of Radiology & Nuclear Medicine, Erasmus MC, P.O. Box 2240, 3000 CA Rotterdam, The Netherlands; 2 Computed Tomography Division, Siemens Healthineers, Forchheim, Germany

**Keywords:** Child, Pediatrics, Tomography, spiral computed, Health physics, Radiation dosage

## Abstract

**Objective:**

To assess the accuracy of a 3D camera for body contour detection in pediatric patient positioning in CT compared with routine manual positioning by radiographers.

**Methods and materials:**

One hundred and ninety-one patients, with and without fixation aid, which underwent CT of the head, thorax, and/or abdomen on a scanner with manual table height selection and with table height suggestion by a 3D camera were retrospectively included. The ideal table height was defined as the position at which the scanner isocenter coincides with the patient’s isocenter. Table heights suggested by the camera and selected by the radiographer were compared with the ideal height.

**Results:**

For pediatric patients without fixation aid like a baby cradle or vacuum cushion and positioned by radiographers, the median (interquartile range) absolute table height deviation in mm was 10.2 (16.8) for abdomen, 16.4 (16.6) for head, 4.1 (5.1) for thorax-abdomen, and 9.7 (9.7) for thorax CT scans. The deviation was less for the 3D camera: 3.1 (4.7) for abdomen, 3.9 (6.3) for head, 2.2 (4.3) for thorax-abdomen, and 4.8 (6.7) for thorax CT scans (*p* < 0.05 for all body parts combined).

**Conclusion:**

A 3D camera for body contour detection allows for automated and more accurate pediatric patient positioning than manual positioning done by radiographers, resulting in overall significantly smaller deviations from the ideal table height. The 3D camera may be also useful in the positioning of patients with fixation aid; however, evaluation of possible improvements in positioning accuracy was limited by the small sample size.

**Key Points:**

*• A 3D camera for body contour detection allows for automated and accurate pediatric patient positioning in CT.*

*• A 3D camera outperformed radiographers in positioning pediatric patients without a fixation aid in CT.*

*• Positioning of pediatric patients with fixation aid was feasible using the 3D camera, but no definite conclusions were drawn regarding the positioning accuracy due to the small sample size.*

## Introduction

Technological developments in computed tomography (CT) enhanced the clinical imaging possibilities in pediatric patients, sparking off a growth in the number of CT scans performed within this population [1, 2]. Over the years, considerable efforts have been made to optimize radiation dose and image quality (IQ) [3, 4]. Several techniques are used to optimize pediatric CT scanning protocols such as automated tube current and tube voltage adaptation, as well as the use of iterative reconstruction techniques [5–8]. For an ideal working of the automatic exposure control (AEC) and to achieve ideal IQ, it is important to position the patient exactly in the center of the CT gantry [9]. Vertical patient positioning is determined by setting the table height. Ideal positioning is defined as the table height at which the patient’s and scanner’s isocenter coincide. Patient positioning lower or higher than the scanner isocenter (i.e., table set too low or too high) affects the patient’s shape and size on a CT scan localizer radiograph, which is of direct effect on the behavior of the AEC. Positioning of pediatric patients is quite challenging, because of the wide variation in body proportions. Furthermore, when they have to be positioned in fixation aids such as a baby cradle or vacuum cushion due to lack of cooperation, it is more difficult to estimate the center of the patient. Recent studies have exhibited the benefits of using a 3D camera and a body contour detection algorithm for the accurate positioning of adult patients, resulting in smaller deviations from the ideal table height compared with manual positioning done by radiographers [10, 11]. The camera algorithm is described in detail in our paper with regard to (adult) patient positioning [11]. It was not yet applicable to pediatric patients due to their different body proportions compared with adults [10, 11]. The algorithm was improved to account for the pose and body proportions of pediatric patients, too. The aim of this study was to determine the accuracy of the new improved version of the algorithm in the positioning of pediatric patients in comparison to manual positioning done by radiographers.

## Materials and methods

### Study design and patient selection

The study was conducted in accordance with the Declaration of Helsinki and international standards of Good Clinical Practice. The medical ethics committee of our hospital waived the need for informed consent. All consecutive pediatric (< 18 years old) patients that underwent a CT examination of the head, thorax, and/or abdomen during routine clinical care in our hospital during a 5-month period from September 2018 to February 2019 were retrospectively included. All scans were performed on a dual-source CT scanner (DSCT) (SOMATOM Drive (software version Syngo CT VA62A), Siemens Healthineers) that was also equipped with a commercially available 3D camera for body contour detection (Siemens Healthineers).

### Manual patient positioning by radiographers

Positioning and scanning of the pediatric patients were done by a team of dedicated CT radiographers as per normal clinical routine. Patients were positioned by the radiographer with the aid of laser beams within the gantry of the CT scanner. A horizontal laser line was projected on the lateral side of the patient and the table height was adjusted by the radiographer so that the laser line was assumed to align the center of the body region to be examined with the scanner isocenter. In our hospital, radiographers are trained to pay special attention to the position of the patient. Scanning was performed after this manual positioning done by radiographers.

### Patient positioning using a 3D camera for body contour detection

**Fig. 1:**
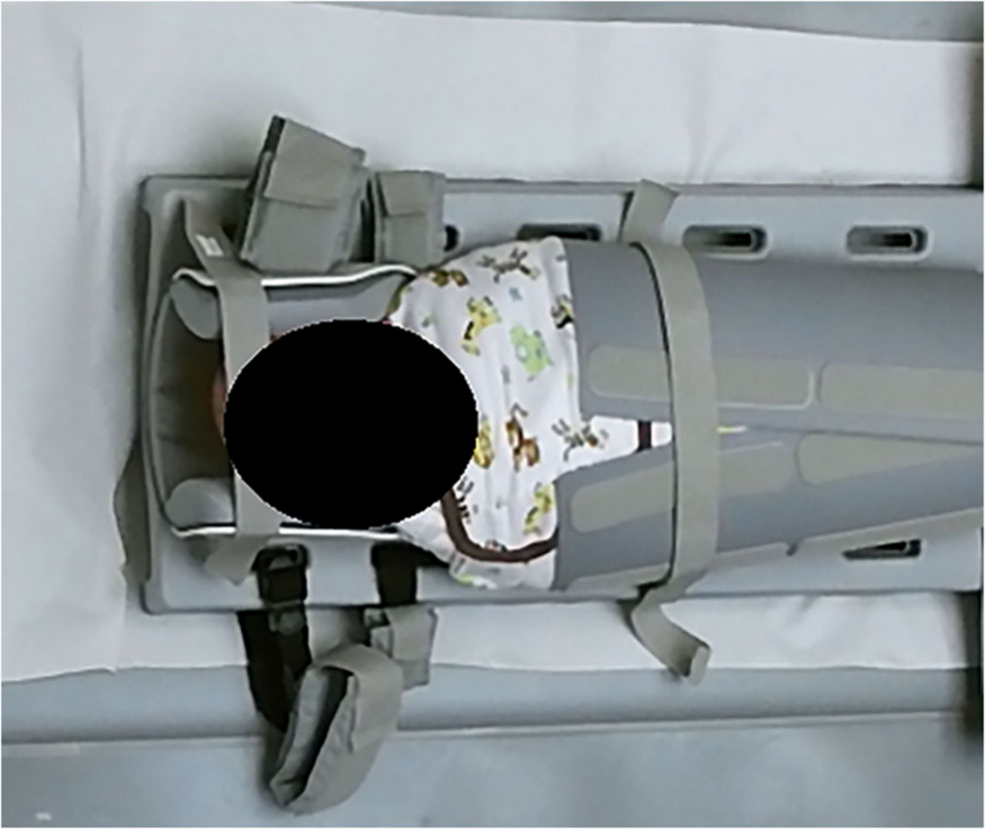
A child under the age of 1 year positioned in a (vendor specific) baby cradle

**Fig. 2:**
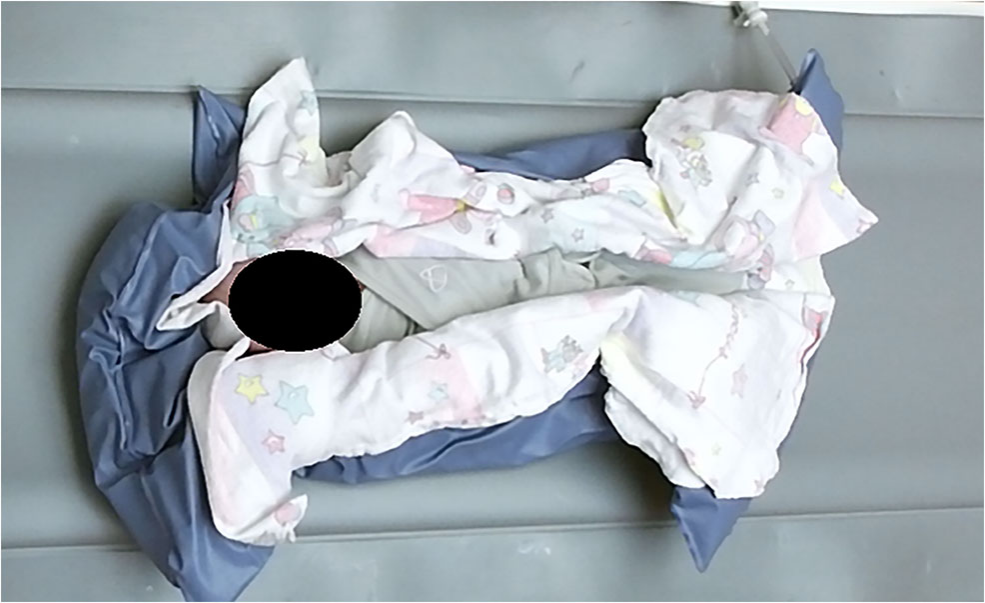
A child under the age of 1 year positioned in a vacuum cushion

3D camera images of children were collected and retrospectively used for the evaluation of the accuracy in patient positioning. The 3D camera is part of the CT system (SOMATOM Drive, Siemens Healthineers) and is attached to the ceiling and in front of the CT scanner, facing down onto the patient table. To start the 3D camera patient positioning process, the radiographer triggers a planning image with the camera when the patient is lying down on the scanner table in the target pose for the CT examination. The camera obtains two images: a color image and a depth image. Each pixel in the depth image describes the distance from the camera to the closest object surface. First, the algorithm detects the pose of the patient and body regions using the depth measurements and the known table position and shape [12]. After selection of the scan range, the ideal table height for the individual patient and the scheduled examination is proposed by the 3D camera such that the isocenter of the selected body region and the scanner isocenter align. Therefore, a virtual patient Avatar is fitted to the camera data in order to cope with areas that cannot be seen by the camera, e.g., through clothing or blankets or to handle positioning aids. The Avatar is a statistical shape model which in the fitting process assumes the pose and body proportions of the patient found in the depth data. The isocenter curve of the Avatar is finally averaged across all slices of the body region selected on the localizer radiograph. If Avatar fitting is not possible, then the isocenter curve is automatically obtained as the geometric center between the camera depth data and the central part of the scanner table. This is the same fallback as described before [11]. For adult patients, the camera images are processed by an algorithm [12], as described in detail before [10, 11]. However, the algorithm installed on the scanner that was used in the (adult) reference study was not optimized for pediatric patients. Therefore, prior to the start of the inclusion for this study, an algorithm training was performed on a separate large dataset of pediatric patients (*n* = 267) to improve the landmark detection and to adjust the Avatar shape model to the different body proportions found in pediatric patients. With the adaptations made, the new version of the algorithm was expected to work for adult and pediatric patients, but was not yet installed at the scanner during the inclusion. Therefore, the algorithm was applied retrospectively without the need of additional data and no user input was required afterwards. By doing so, offline system performance is equal to the real-world situation. When the algorithm was not able to fit the Avatar, the regular and automatic fallback was applied for patients positioned with and without a fixation aid, such as a baby cradle (Fig. 1) or a vacuum cushion (Fig. 2). The algorithm is currently not commercially available.

### Calculation of patient positioning accuracy

CT image reconstructions with a slice thickness of 3.0 mm and a reconstruction increment of 3.0 mm were used. The reconstructed field-of-view included the entire skin surface. The skin surface was extracted from the CT data in each axial slice and used to calculate the middle of the patient in the anterior-posterior direction. These values were averaged over all slices along the z-axis, providing the patient isocenter, to determine the ideal table height as described in detail before [11]. Accuracy in patient positioning is demonstrated as the difference between this ideal table height and the table height proposed by the camera algorithm or the radiographer. The accuracy is expressed as a single and absolute value in millimeter. The distribution of patients with a table height deviation lower or higher than the ideal table height is expressed as positive or negative numbers, respectively.

### Exclusion of scans

Cases with obvious patient movement or repositioning between the body contour detection by the 3D camera and the CT scan or with large items blocking the camera sight were excluded.

### Statistical analyses

Significant differences in patient positioning between the radiographers and the 3D camera were analyzed by means of normality and a nonparametric test. The absolute table height deviation is a continuous paired variable reported as median (interquartile range (IQR)), calculated with Microsoft Excel (Microsoft Office Professional Plus 2016). Data distribution was tested with the Shapiro-Wilk test. Wilcoxon signed-rank test was used for comparison of the absolute table height deviation for the different body regions within and between the camera and radiographer group. A two-sided *p* value of < 0.05 was considered statistically significant. Statistical analyses were performed using SPSS (version 25, IBM Corp). Continuous measures of absolute table height deviation (mm) were calculated and evaluated with Microsoft Excel (Microsoft Office Professional Plus 2016). A post hoc power analysis was performed with G*Power (version 3.1.9.6) for the patients positioned with a fixation aid to determine the effect size, given the power to be achieved (80%) and the sample size available [13, 14]. The purpose of this test is to estimate the smallest possible difference in patient positioning accuracy between the 3D camera and the radiographer that can be detected in this study.

## Results

### Patient groups

After exclusion of sixteen scans from the analysis due to patient repositioning after body contour detection by the 3D camera or because of large objects blocking the camera sight, one hundred and ninety-one scans were available for analysis. Of which, 149 pediatric patients were without and 42 patients were with a baby cradle or vacuum cushion. Within the group without fixation aids, the median age (IQR) was 11 years (6) and ranged between 3 months and 17 years old. For patients positioned in the baby cradle or the vacuum cushion, the median age (IQR) was 0.8 years (1.4) and ranged between 1 day and 6 years.

### Patient positioning accuracy of radiographers

**Fig. 3:**
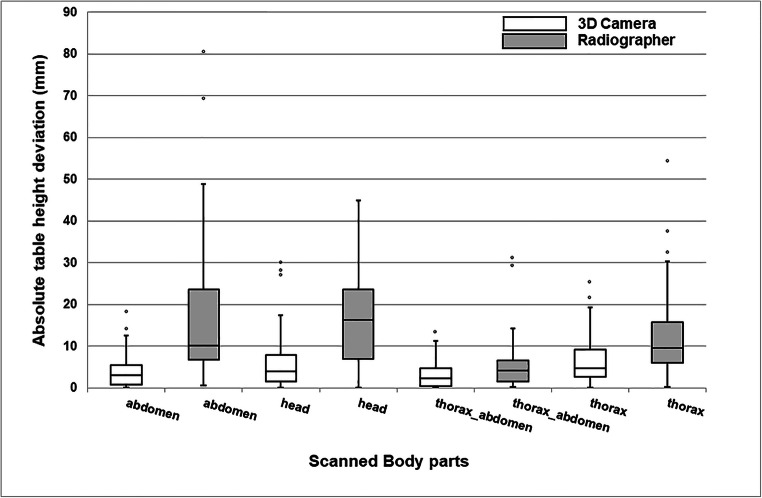
Box-and-whisker plots of patient positioning performance of all different body parts separately for the radiographers and the 3D camera for pediatrics positioned without fixation aid. The median (horizontal line within box), interquartile range (box), and nonoutlier range (whiskers) which is defined as 1.5 times interquartile range (i.e., 25—75%). The largest deviations from the scanner isocenter, outside the nonoutlier range, are plotted as open dots

Within the group without fixation aids, median (IQR) absolute table height deviation was 10.2 (16.8) for abdomen, 16.4 (16.6) for head, 4.1 (5.1) for thorax-abdomen, and 9.7 (9.7) mm for thorax CT scans positioned by radiographers (Fig. 3; Table 1). A total of 41 (28%) patients were positioned higher than the scanner isocenter. The majority of patients were positioned lower than the scanner isocenter (Table 1).

**Table 1:** Pediatric patient positioning without a baby cradle or vacuum cushion: comparison of table height deviation for radiographers and 3D camera

Body part	Abdomen	Head	Thorax-abdomen	Thorax	All body parts combined
Total number of patients without a baby cradle or vacuum cushion	22 (15%)	46 (31%)	14 (9%)	67 (45%)	149 (100%)
Table height determined by radiographers					
Median of absolute table height deviation, mm	10.2 [16.8]	16.4 [16.6]	4.1 [5.1]	9.7 [9.7]	10.3 [12.6]
Patients positioned higher than isocenter, *n (%)*	5 (23%)	15 (33%)	2 (14%)	19 (28%)	41 (28%)
Patients positioned lower than isocenter, *n* (%)	17 (77%)	31 (67%)	12 (86%)	48 (72%)	108 (72%)
Largest deviation, mm (age in years)	80.5 (13 years)	44.9 (3 years)	31.1 (12 years)	54.3 (11 years)	
Table height determined by 3D camera					
Median of absolute table height deviation, mm	3.1 [4.7]	3.9 [6.3]	2.2 [4.3]	4.8 [6.7]	3.7 [5.8]
Patients positioned higher than isocenter, *n* (%)	8 (36%)	22 (48%)	6 (43%)	33 (49%)	69 (46%)
Patients positioned lower than isocenter, *n* (%)	14 (64%)	24 (52%)	8 (57%)	34 (51%)	80 (54%)
Largest deviation, mm (age in years)	18.2 (15 years)	− 30.1 (10 years)	− 13.5 (13 years)	− 25.4 (3 years)	
*p* value median absolute table height deviation (3D camera versus radiographer)	< 0.005	< 0.005	0.064	< 0.005	< 0.005

Data are numbers (%) and median [interquartile range]

Negative deviation numbers: patient positioned higher than isocenter

Positive deviation numbers: patient positioned lower than isocenter

For the 42 patients positioned in the baby cradle or the vacuum cushion, the median (IQR) absolute table height deviation was 8.7 (1.1) for abdomen, 9.1 (12.9) for head, 8.0 (3.1) for thorax-abdomen, and 15.3 (15.8) mm for thorax CT scans (Table 2). A total of 12 (29%) patients were positioned higher than the scanner isocenter. Within this group, the majority of patients were also positioned lower than the scanner isocenter (Table 2).

**Table 2:** Pediatric patient positioning with a baby cradle or vacuum cushion: comparison of table height deviation for radiographers and 3D camera

Body part	Abdomen	Head	Thorax-abdomen	Thorax	All body parts combined
Total number of patients with a baby cradle or vacuum cushion	3 (7%)	20 (48%)	2 (5%)	17 (40%)	42 (100%)
Table height determined by radiographers					
Median of absolute table height deviation, mm	8.7 [1.1]	9.1 [12.9]	8.0 [3.1]	15.3 [15.8]	9.2 [13.7]
Patients positioned higher than isocenter, *n* (%)	1 (33%)	4 (15%)	1 (50%)	6 (41%)	12 (29%)
Patients positioned lower than isocenter, *n* (%)	2 (67%)	17 (85%)	1 (50%)	10 (59%)	30 (71%)
Largest deviation, mm (age in months)	10.3 (12 M)	− 32.8 (3 M)	11.1 (2 M)	− 42.4 (48 M)	
Table height determined by 3D camera					
Median of absolute table height deviation, mm	10.8 [8.3]	10.2 [15.3]	17.4 [16.0]	15.2 [15.0]	10.9 [16.6]
Patients positioned higher than isocenter, *n* (%)	2 (67%)	18 (85%)	1 (50%)	7 (47%)	28 (67%)
Patients positioned lower than isocenter, *n* (%)	1 (33%)	3 (15%)	1 (50%)	9 (53%)	14 (33%)
Largest deviation, mm (age in months)	− 23.3 (72 M)	− 67.1 (23 M)	33.4 (2 M)	− 46.4 (5 M)	
*p* value median absolute table height deviation (3D camera versus radiographer)	0.593	0.167	0.655	0.850	0.105

Data are numbers (%) and median [interquartile range]

Negative deviation numbers: patient positioned higher than isocenter

Positive deviation numbers: patient positioned lower than isocenter

### Patient positioning accuracy of 3D camera

Within the group without fixation aids, median (IQR) absolute table height deviation in millimeter was 3.1 (4.7) for abdomen, 3.9 (6.3) for head, 2.2 (4.3) for thorax-abdomen, and 4.8 (6.7) for thorax CT scans (Fig. 3; Table 1). A small majority of the patients were positioned lower than the scanner isocenter (Table 1).

Within the patient group positioned in the baby cradle or vacuum cushion, the median (IQR) absolute table height deviation was 10.8 (8.3) for abdomen, 10.2 (15.3) for head, 17.4 (16.0) for thorax-abdomen, and 15.2 (15.0) mm for thorax CT scans. Within this group, the majority of patients were also positioned lower than the scanner isocenter (Table 2). An Avatar could be used in three out of nine cases when a patient was positioned in a baby cradle and the fallback had to be applied for all cases positioned in a vacuum cushion.

### Comparison between radiographer and 3D camera

**Fig. 4:**
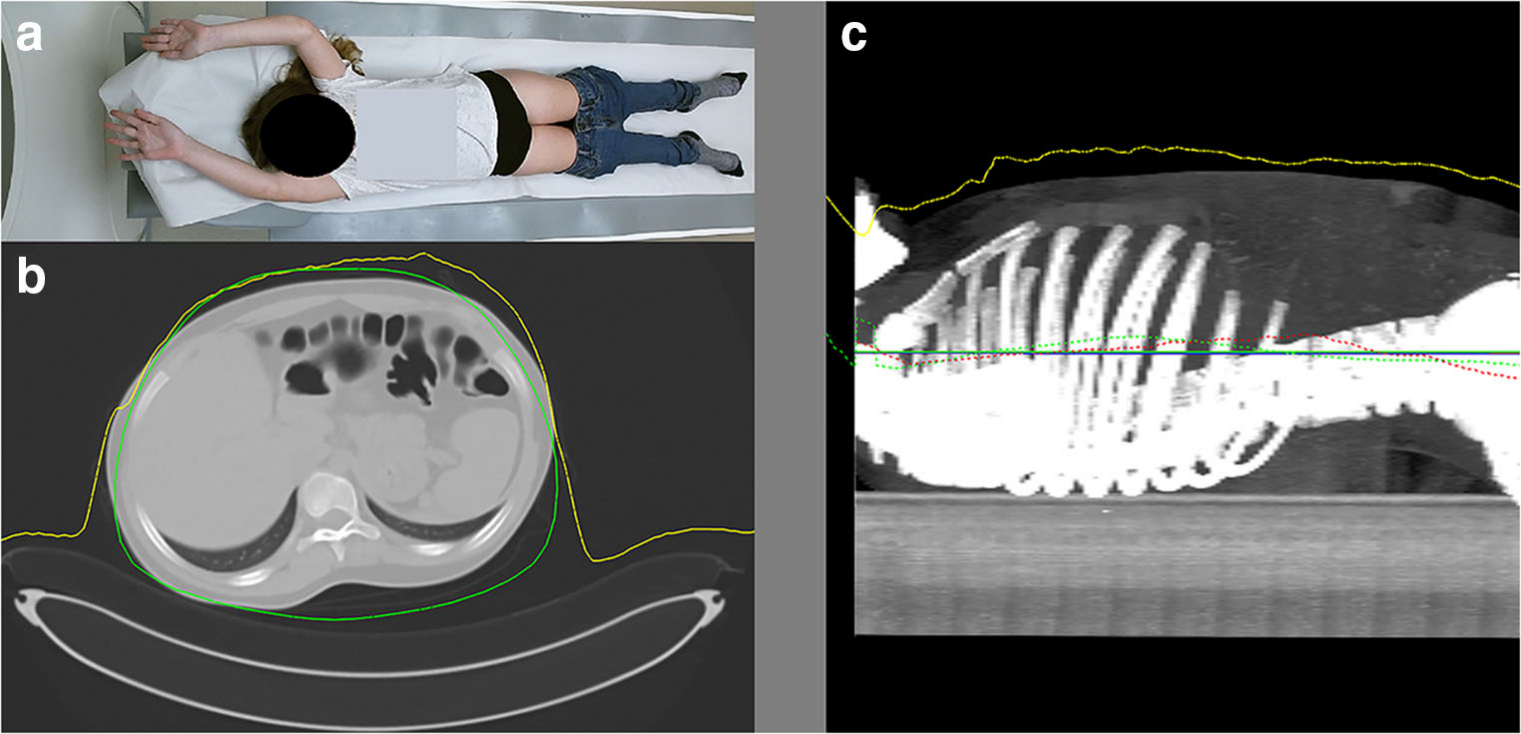
Case presentation of a 12-year-old child. **a** Color image taken by the 3D camera system. **b** Axial image of the abdomen with depth measurements (yellow line) by the 3D camera and the body contour (green) estimated by the algorithm. **c** Sagittal image of the thorax-upper abdomen with patient positioning accuracy: blue horizontal line: patient isocenter estimated by the radiographer, green horizontal line: average patient isocenter estimated by the camera, green dotted line: Avatar isocenter curve, red horizontal line: average patient isocenter (ideal table height), red dotted line: patient isocenter per axial cross-section, yellow line: depth measurements. The red, green, and blue straight lines are hard to distinguish from each other due to almost similar values as the ideal table height

For patients positioned without the baby cradle or vacuum cushion, the median absolute table height deviations were higher for all four body parts when positioned by the radiographer compared with table height suggestion by the 3D camera (Table 1). For each of the four body parts, the largest deviation from the ideal table height was also higher for patients positioned by the radiographer. The largest deviation was 80.5 mm for an abdominal scan with the pediatric patient positioned lower than the isocenter by the radiographer. For the 3D camera, the largest deviation was 30.1 mm. Patient positioning accuracy for the 3D camera system and the radiographers differed significantly for all body parts (*p* < 0.005) with the exception of thorax-abdomen scans (*p* = 0.064). Figure 4 shows a case presentation with small deviations from the ideal table height of 0.13 mm and − 0.82 mm for the 3D camera and radiographer, respectively.

For patients positioned with the baby cradle and vacuum cushion, the deviation from the ideal table height by the 3D camera was up to 46.4 mm for a thoracic scan (Table 2). For the radiographer, the largest deviation was 42.4 mm below the isocenter. Difference between patient positioning accuracy of the 3D camera system and the radiographers was not significant (Table 2). For both groups, the mean and standard deviation (SD) of the table height deviation for all body parts combined (*n* = 42) was calculated for the post hoc power analysis. The mean (SD) was 17.1 mm (15.6) and 12.2 mm (9.4) for the 3D camera and radiographers group, respectively, with a correlation coefficient between groups of 0.2. The effect size or Cohen’s *d* corresponding with a power of 80% was 0.45. This is equivalent to a difference of 8.2 mm between camera and radiographers.

**Fig. 5:**
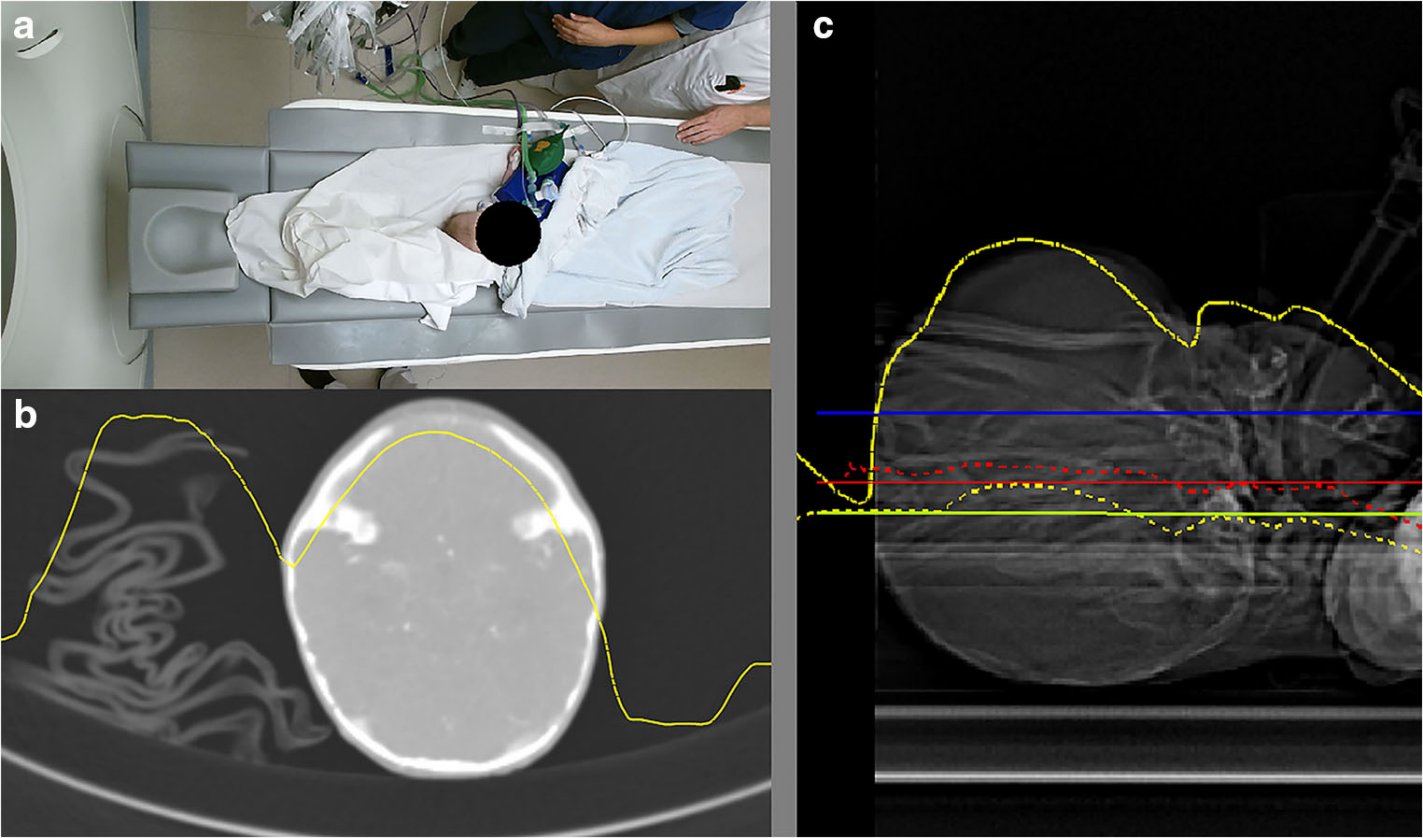
Case presentation of a 3-month-old child wrapped in a blanket with a breathing support and wires where accurate positioning is challenging for both the 3D camera and the radiographers. **a** Color image taken by the 3D camera system. **b** Axial image of the brain with depth measurements (yellow line) by the 3D camera. **c** Sagittal image of the head with patient positioning accuracy: blue horizontal line: patient isocenter estimated by the radiographer, green horizontal line: algorithm isocenter estimated by the camera, green dotted line: algorithm isocenter curve, red horizontal line: average patient isocenter (ideal table height), red dotted line: patient isocenter per axial cross-section, yellow line: depth measurements

Positioning of young patients with lots of cabling, vital monitoring devices, or blankets was challenging for accurate positioning by both the 3D camera and the radiographers. Such a case presentation is presented in Fig. 5 with an ideal table height deviation of − 10.3 mm and 23.8 mm for the 3D camera and radiographer, respectively.

## Discussion

We assessed the accuracy of pediatric patient positioning with the aid of a body contour detection system (3D camera) and compared it with manual positioning by radiographers. We found that positioning with the 3D camera of pediatric patients without a fixation aid allows for more accurate patient positioning than manual positioning by radiographers. This outcome is similar to the findings in adult patients [10, 11]. Differences in positioning accuracy between the 3D camera and radiographers were not statistically significant for patients positioned in a baby cradle or a vacuum cushion. In virtually all cases of infants placed in fixation aids, like a baby cradle (Fig. 1) or a vacuum cushion (Fig. 2), it was not possible to fit a patient Avatar due to the small body size and the large occlusions. Instead, the fallback described above was applied, where the isocenter is directly estimated as geometric mean between the depth measurements and the table. This approach introduces a deviation, because these fixation aids add a considerable layer between patient and table, which in the absence of the Avatar is wrongly attributed to the patient, leading to an overestimation of patient size. Nevertheless, positioning of patients in a fixation aid seems feasible with a 3D camera. Small performance differences between camera and radiographers could not be detected due to the limited number of patients included. However, post hoc power analysis showed that the performance difference was not larger than 8.2 mm; otherwise, this would have been noted given our sample size. Thus, the fallback routine facilitates automatic positioning of a pediatric patient while keeping possible differences with a well-trained radiographer below 10 mm on average.

However, the deviation from the ideal table height could be reduced by taking the positioning devices into account. Therefore, applying an intermediate step consisting of the detection of the presence of a fixation aid like a baby cradle and a vacuum cushion (open and closed) might be of use and may be considered for further research. After detection of such aid, a correction for the thickness of such an aid can be applied to the geometric isocenter for these specific cases. The correction can be determined upfront by estimating the mean error for the vacuum cushion and by accounting for the fairly constant thickness of the baby cradle.

The two main challenges for the algorithm are the small size of the patients that are positioned with such aids and the large degree of occlusions introduced by the aids. Given a large amount of 3D camera training images, probably we could reliably fit the patient Avatar also under these circumstances. Then, as usual for cases without a baby cradle or a vacuum cushion, it would be possible to compute the center of the patient Avatar and naturally exclude additional layers such as blankets or fixation aids. The Avatar fitting was only possible in three out of nine cases when patients were positioned in a baby cradle and the fallback had to be applied in all cases when positioned in a vacuum cushion. Therefore, further work on the development with additional training data might improve the algorithm even further to reliably obtain a patient isocenter when patients are positioned in fixation aids like the baby cradle and vacuum cushion.

The 3D camera is able to assist the radiographer in positioning of pediatric patients, especially in cases without fixation aid. It should be emphasized that radiographers will continue to play an important role in patient positioning by patient guidance and verification of the table height suggested by the 3D camera, especially when fixation aids are used.

Studies demonstrated a significant impact on radiation dose and image quality when a pediatric patient is not properly positioned in the scanner isocenter [15, 16]. In those studies, an anthropomorphic head, thorax, and/or abdomen simulating on a 5-year-old child was used. Organ doses and noise differences with several vertical table height deviations were compared with organ dose and noise levels at the scanner isocenter/center position. A noise increase of up to 45% in chest scans relative to the center position was demonstrated for table positions in the highest (+ 54 mm) and lowest (− 60 mm) vertical positions and a breast dose increase of up to 16% with 40 mm lower vertical position [16]. Although the absolute table height deviations in our study were not always that high, maximum deviation values were high, especially with radiographers (Tables 1 and 2). Relative breast dose increase was considered to be 7% lower with 20 mm vertical lower positioning compared with the 40 mm lower position. This vertical positions are comparable to the values of the largest deviations between the 3D camera and radiographers in our study. With the tendency to position pediatric patients more often lower than the ideal table height, the noise would increase. With less extreme deviations from the ideal table height that can be obtained with the 3D camera (Tables 1 and 2), both the radiation dose and the image quality will be more constant. The same applies for organ radiation doses and image noise in head and abdominal CT [15]. Large vertical table height deviation was of substantial influence on radiation dose and image noise, where the impact of these deviations depends on the body region and location of individual organs within the body [15]. However, accurate and less deviations from the ideal table height are required to consolidate image quality and radiation dose. Our results were obtained in an academic facility with highly trained radiographers. It is conceivable that both the median and maximum values of deviation from the ideal positioning would be even larger when the study was obtained in a hospital without dedicated training in pediatric CT scanning.

There are limitations to this study that require considerations. For the purpose of the analysis, the algorithm used the actually scanned range to calculate the isocenter. This differs from routine operation of the camera system, whereby the algorithm uses the scan range that is defined on the planning image (=color photograph taken by the camera) prior to obtaining the localizer radiograph and scanning the patient. Consequently, the suggested ideal table height by the 3D camera based on the planned scan range may differ from the suggested table height based on the actual scan range. Nevertheless, our results demonstrate the accuracy when a 3D camera is used properly and the selected body region on the localizer radiograph and the actual scan range are the same.

In conclusion, a 3D camera for body contour detection allows for accurate pediatric patient positioning in CT. The 3D camera is able to assist the radiographer in positioning of pediatric patients, especially in cases without fixation aid. Positioning of patients in a fixation aid is feasible with a 3D camera, but evaluation of possible improvements in positioning accuracy was limited by the small sample size.

## Abbreviations

AEC: Automatic exposure control
CT: Computed tomography
DSCT: Dual-source computed tomography
IQ: Image quality
IQR: Interquartile range
SD: Standard deviation
